# Assessment of Dermally Bioaccessible Elements by Sweat-Simulated Extraction: Analytical Approach and Application to Tattoo Inks

**DOI:** 10.3390/molecules31111804

**Published:** 2026-05-24

**Authors:** Carmela Protano, Arianna Antonucci, Maria Luisa Astolfi

**Affiliations:** 1 Department of Benessere, Salute e Sostenibilità Ambientale, Sapienza University of Rome, 00185 Rome, Italy; carmela.protano@uniroma1.it (C.P.); arianna.antonucci@uniroma1.it (A.A.); 2 Department of Chemistry, Sapienza University of Rome, 00185 Rome, Italy

**Keywords:** artificial sweat extraction, elemental analysis, ICP-MS, metal leaching, sample preparation, tattoo inks

## Abstract

The determination of soluble elemental contaminants in tattoo inks is challenged by the lack of standardized extraction procedures, limiting the comparability of analytical results and the assessment of exposure-relevant fractions under the European REACH framework. In this study, artificial sweat extraction was applied as a mild and physiologically relevant approach to evaluate elements potentially released from tattoo inks under sweat-simulated skin-contact conditions. Seventy-eight commercial tattoo inks of different colors were extracted with artificial sweat at 37 °C for 1 h and analyzed by inductively coupled plasma mass spectrometry. Optimization of collision/reaction cell conditions, dilution strategy, and internal standard correction effectively reduced matrix-related interferences caused by the high salt and chloride content of artificial sweat, ensuring reliable quantification. Matrix-matched calibration was required due to significant signal suppression for several analytes. Method accuracy and precision, assessed using NIST 1643f and spiked samples, were generally satisfactory. Elemental release showed marked color-dependent trends, particularly for Cu, Zn, Ba, Al, Ga, Si, Sr, and Zr, reflecting differences in pigment composition and formulation. Soluble Ba, Cu, and Zn remained below EU regulatory limits. While total digestion remains essential for complete characterization, the proposed methodology provides a simple and transferable tool for exposure-oriented assessment of potentially bioaccessible elements in tattoo inks.

## 1. Introduction

The practice of tattooing has gained widespread popularity across diverse age groups in recent decades, especially among adolescents and young adults [1,2]. Epidemiological surveys indicate that a substantial portion of individuals aged 18–35 years in Western countries have at least one tattoo, often acquired during formative years when long term exposure implications may be particularly relevant [1,3]. This pervasive use, combined with the permanence of tattoo inks in the dermal layer, raises important public health questions, as the skin may be exposed chronically to chemical constituents embedded within these complex formulations [4].

Tattoo inks are typically mixtures of inorganic pigments, organic colorants, solvents, dispersants, and additives designed to impart desired color and stability [3]. While organic pigments contribute to hue, inorganic components frequently consist of metal-based oxides and salts, which are used to confer specific chromatic characteristics, opacity, and long-term stability to tattoo inks, with Fe oxide dominating darker shades and Ti dioxide used for lighter hues [5]. However, trace levels of potentially toxic elements have been detected in commercial tattoo inks, either as intrinsic constituents of pigment matrices or as incidental contaminants from production and processing [6]. Metals of toxicological concern, including Cd, Co, Cr, Cu, Ni, and Pb, have been identified across a range of ink colors and brands, with variations that appear related to pigment chemistry and manufacturing practices [6,7]. The toxicological profiles of these metals are well established. Chromium as Cr(VI) is a potent contact sensitizer and recognized carcinogen in occupational settings, with evidence linking dermal exposure to allergic dermatitis [7]. Nickel is among the most common contact allergens worldwide and is subject to regulatory limits in items intended for prolonged skin contact due to sensitization risk [8]. Lead and Cd are classified as human toxicants with neurotoxic, reproductive, and systemic effects and are strictly regulated in environmental and consumer contexts because even low-level chronic exposure is associated with health detriments [9,10]. Although essential at trace levels for physiological processes, certain metals present in tattoo inks, such as Cu and Co, may exert adverse effects under elevated exposures. Copper species, particularly CuO nanoparticles, have been shown to cause dose-dependent cytotoxicity in human skin cells and to elicit inflammatory responses in ex vivo skin models, while cobalt is recognized as a potential allergen and toxicant at higher concentrations [11,12]. Given the permanence of tattoos and the potential longevity of ink deposition in dermal tissue, even low-level exposures may have health relevance over time. Importantly, tattoos are now increasingly common among adolescents, highlighting the necessity of assessing exposure early in life. To manage these risks, the European Council and subsequent regulations have established limits for hazardous elements in tattoo inks. Specifically, elements regulated for total content include As, Cd, Co, Cr(VI), Hg, Ni, Pb, Sb, and Sn, while elements regulated for the soluble fraction include Ba, Cu, and Zn [13,14]. These measures aim to reduce potential adverse effects by controlling both the total metal load and the bioaccessible fraction in tattoo inks.

However, a major limitation of existing regulatory frameworks, such as the European Council Resolution ResAP(2008)1 [13], is the lack of clearly defined analytical procedures, including the choice of extraction medium, sample mass, and exposure time [15]. As observed for other consumer products, such as toys or jewellery, these parameters critically influence the measured soluble metal fraction, contributing to inconsistencies in reported data and complicating both risk assessment and regulatory enforcement. Although more recent regulations, such as the European Union Registration, Evaluation, Authorization and Restriction of Chemicals (REACH, Regulation (EU) 2020/2081) [14], have introduced legally binding restrictions for certain substances in tattoo inks, similar challenges persist when analytical methods are not explicitly specified.

In this context, analytical studies investigating metal release from tattoo inks under simulated physiological conditions remain scarce, emphasizing the need for standardized protocols to reliably determine the bioaccessible fraction of metals [6].

Commonly applied instrumental analytical techniques include inductively coupled plasma optical emission spectrometry (ICP-OES) [16], graphite furnace atomic absorption spectrometry (GF-AAS), and flame atomic absorption spectrometry (FAAS) [17]. Inductively coupled plasma mass spectrometry (ICP-MS) is also widely used and has been applied in single-particle mode (SP-ICP-MS) for the detection and characterization of metal-based micro- and nanoparticles in tattoo inks [11,18,19,20]. ICP-MS is generally preferred in tattoo ink studies due to its ultra-trace sensitivity, broad multi-element capability, and wide dynamic range, allowing simultaneous quantification of major and trace constituents [20]. Additionally, ICP-MS offers low limits of detection, allowing the measurement of trace levels of critical toxic metals such as Ni, Cd, and Pb, which may be present at very low concentrations after extraction. High-resolution or collision/reaction cell (CRC) ICP-MS further mitigates spectral interferences from complex matrices, improving analytical accuracy [21,22].

Studies utilizing comprehensive elemental profiling have revealed that the distribution of metals in tattoo inks is influenced by pigment composition and color family [19,23,24]. For example, Fe- and Ti-based pigments common in darker inks may be accompanied by trace levels of Mn or V, while Cd-sulfide-based pigments historically used in yellow and some red tattoo inks raise concerns due to cadmium’s toxicity and potential for bioaccumulation [6,25]. Copper-containing phthalocyanine pigments, widely used to achieve blue and green hues in tattoo inks, have been identified as major sources of total Cu content in these products; however, due to the strong binding of Cu within the phthalocyanine macrocycle, only a fraction of this Cu is released in bioavailable form, making the distinction between total and soluble Cu critical for dermal exposure assessments [26].

This study investigates the elemental composition of a large set of commercial tattoo inks, focusing on the fraction of elements released under artificial sweat extraction. This approach provides a practical proxy for estimating the bioaccessible fraction of metals, offering useful indications of potential dermal exposure and enabling comparisons across studies. Although tattoos are deposited in the dermis and the method does not fully replicate this environment, it can contribute to improving understanding of metal bioaccessibility and guide future research on potential health risks.

In addition, particular attention is devoted to the evaluation and validation of the analytical method, including calibration characteristics, background contribution (BEC), limits of detection and quantification (LOD and LOQ), as well as accuracy and precision. These aspects are still poorly addressed in studies dealing with the elemental composition of tattoo inks, especially with regard to the soluble fraction, which remains scarcely investigated in the available literature. By systematically assessing these analytical performance parameters, the present work aims to provide a more robust and reliable framework for the determination of bioaccessible metals in tattoo inks.

## 2. Results and Discussion

### 2.1. Optimization of Experimental Parameters

The combined application of extract dilution, internal standard correction (ISC), and CRC technology effectively mitigated matrix effects associated with the artificial sweat, resulting in improved instrumental stability and reliable, reproducible elemental quantification.

#### 2.1.1. Optimization of Collision Cell Gas and Flow Rate

When chloride ions are present in the solutions analyzed by ICP-MS, the formation of polyatomic species such as ^40^Ar^35^Cl^+^ can cause severe spectral overlap with the ^75^As^+^ signal, leading to positively biased results. In contrast, elements such as Cd (^111^Cd^+^) and Pb (^208^Pb^+^) are comparatively less affected by chloride-based polyatomic interferences under standard operating conditions. Given the high chloride content of artificial sweat, the use of CRC technology was therefore essential to mitigate these spectral interferences [27].

Both He and H_2_ cell gases were evaluated to improve analytical selectivity and sensitivity. Helium mode operates predominantly through kinetic energy discrimination, effectively attenuating larger polyatomic ions formed in the plasma, whereas H_2_ acts as a reaction gas, promoting chemical reactions that selectively remove interfering species. In the present study, the combined use of H_2_ and He proved effective for elements particularly susceptible to polyatomic interferences, including As, Cr, Fe, Mn, Se, and V. Optimal performance was achieved using H_2_ at a flow rate of 70 mL min^−1^ and He at 30 mL min^−1^ (Table 1), resulting in a significant reduction in background signals and improved signal-to-noise ratios.

Under these optimized conditions, spectral interferences associated with argon–chloride and sodium-based polyatomic ions (e.g., ^40^Ar^37^Cl^+^, ^23^Na^52^Cr^+^) were efficiently suppressed, enabling accurate determination of target elements in the artificial sweat extracts. The selected CRC settings also contributed to enhanced instrumental stability and reproducibility across analytical sequences, particularly when combined with appropriate dilution and internal standard correction strategies.

#### 2.1.2. Dilution Ratio and Matrix Effects

Dilution represents the first strategy for mitigating matrix effects in ICP-MS, although it inevitably involves a trade-off with analytical sensitivity. In the present study, dilution of the artificial sweat extracts was necessary to ensure stable aerosol generation and efficient nebulization. Artificial sweat contains high concentrations of easily ionizable elements such as Na and K, together with chloride ions, which can alter plasma ionization conditions and promote the formation of polyatomic species, leading to both spectral and non-spectral interferences. Under the selected conditions, the extracts were diluted 1:10 with 2% (*v*/*v*) HNO_3_ prior to ICP-MS analysis. This dilution reduced the total dissolved solids (TDS) introduced into the plasma and limited matrix-related interferences associated with the high salt and chloride content of the extraction medium, while preventing salt deposition in the sample introduction system in accordance with instrument manufacturer recommendations. Elevated chloride concentrations may generate polyatomic ions such as ^40^Ar^35^Cl^+^, which interferes with the determination of ^75^As^+^, as well as other argon- or sodium-based species (e.g., ^40^Ar^37^Cl^+^ and ^23^Na^52^Cr^+^) that can affect the accurate quantification of elements including Se (^78^Se^+^, ^80^Se^+^), V (^51^V^+^), and Cr (^52^Cr^+^). Such spectral overlaps may increase the background signal or produce apparent signal enhancement, ultimately compromising analytical accuracy. Nevertheless, under the adopted dilution conditions the responses of the internal standards remained close to unity, indicating stable plasma conditions and effective compensation for signal fluctuations.

Matrix effects were further evaluated by comparing the slopes of calibration curves prepared in 2% (*v*/*v*) HNO_3_ and in artificial sweat (1:10). The magnitude of matrix-induced variation was expressed as percentage bias (Δ%), calculated as the relative difference between matrix-matched and aqueous calibration slopes (Table 2). Most elements exhibited negative Δ% values, indicating general signal suppression in the artificial sweat matrix relative to the acidic solution, with values typically ranging between −20% and −55%. This behavior can be attributed to the composition of artificial sweat, which contains relatively high concentrations of alkali and alkaline-earth ions (e.g., Na^+^, K^+^, Cl^−^) together with organic components such as lactate and urea. These species may influence aerosol formation, transport efficiency, and plasma ionization processes, leading to variations in analyte signal intensity. Similar matrix-induced signal suppression has been widely reported for saline and biological matrices analyzed by ICP-based techniques [27,28].

For several elements, including Al, B, Be, Cd, Co, Cr, Cu, Fe, Ga, K, Li, Mg, Mn, Nb, Ni, P, Rb, Sb, Ti, U, V, and Zr, Δ% values ranged between −35% and −55%, indicating pronounced signal suppression consistent with plasma loading and ionization competition effects in the presence of high concentrations of easily ionizable elements. Other analytes, such as Ce, Dy, Gd, La, Mo, Pr, Si, Sn, Sr, Tb, Te, W, and Zn, showed more moderate variations (approximately −15% to −30%), whereas Tl, Se, Pb, and Ba exhibited minimal differences between calibration media (|Δ%| ≤ 10%), suggesting negligible matrix influence under the adopted conditions. A slight signal enhancement was observed for Nd (Δ% = +11%), likely related to local variations in plasma ionization conditions. The most pronounced suppression was observed for As (Δ% = −70%), consistent with the well-known analytical challenges associated with As determination by ICP-based techniques, including relatively low ionization efficiency and susceptibility to spectral and non-spectral interferences [27]. Background equivalent concentrations were also evaluated to assess the contribution of background signal under the different calibration conditions (Table 2). The BEC values were generally higher in the artificial sweat matrix compared with the aqueous calibration medium, suggesting an increased background signal likely associated with the high salt content and the formation of matrix-related polyatomic species. However, the magnitude of matrix effects was more clearly evidenced by the variation of calibration slopes, which showed systematic signal suppression for most analytes. This behavior is consistent with plasma loading and ionization competition effects commonly observed in saline matrices analyzed by ICP-MS [27,28]. In particular, elements such as P, K and Si exhibited markedly elevated BEC values in the artificial sweat solution, in some cases approaching or exceeding the calibration range. Sodium showed an even more pronounced effect: due to the very high endogenous Na concentration in artificial sweat, the analytical signal was dominated by the matrix background, and no measurable signal increase was observed across the investigated calibration range (“signal not increasing”). Consequently, a reliable matrix-matched calibration curve for Na could not be established under the adopted conditions. This behavior is consistent with the known limitations associated with the determination of major, easily ionizable elements by ICP-MS in highly saline matrices, where plasma loading effects and high background signals can compromise calibration linearity. Overall, these effects resulted in significantly increased LOD and LOQ values and limited analytical reliability at low concentrations. Therefore, Na, P, K, and Si were excluded from quantitative evaluation and considered only qualitatively.

To minimize matrix-induced variations, all measurements were performed using internal standardization with Sc^45^, Y^89^, Rh^103^, In^115^, and Th^232^, selected to cover a broad mass range and compensate for variations in sample introduction efficiency, plasma stability, and instrumental drift. Although internal standardization improved signal stability, residual differences between calibration slopes obtained in 2% HNO_3_ and artificial sweat were still observed for several elements. Consequently, accurate quantification was achieved using matrix-matched calibration curves prepared in artificial sweat, an approach commonly adopted when residual matrix effects persist despite internal standard correction [27,28].

#### 2.1.3. Accuracy Validation

For elements with certified concentrations in the reference material, method accuracy was evaluated by calculating the percentage bias relative to the certified values. For elements not included in the certificate, accuracy was assessed through spike-and-recovery experiments at four concentration levels representative of the working range (Table 3).

For trace elemental analysis in the artificial sweat matrix, recoveries between approximately 80–120% and RSD values generally below 15% were considered acceptable. Under these criteria, most trace elements showed satisfactory accuracy and precision, particularly at concentration levels representative of the analyzed tattoo ink extracts. For certified elements, percentage bias values were generally within acceptable limits, confirming the trueness of the method under matrix-matched conditions. Exceptions included K, Na, Mo, Se, Te, and Fe, which exhibited bias values exceeding 20%, likely due to residual matrix effects, elevated background signals, or proximity to the quantification limits.

For non-certified elements, recoveries above 120% (e.g., Al, As, Ba, Cr, Cu, K, Mo, P, Na, Si, Ti, V, and Zn) were mainly observed at the lowest spike levels and were associated with high background contributions and BEC-related signal enhancement. This effect was particularly pronounced for elements with high LOQ values (e.g., Na, P, and K), where the analytical signal is strongly influenced by the matrix background, leading to apparent overestimation at low fortification levels. Similar behavior was also observed for Cr and V, for which elevated recoveries and higher RSD values occurred primarily at the lowest spike concentrations, whereas recoveries and precision improved substantially at medium and high fortification levels.

Specifically, for Na and K, a reliable accuracy assessment was not feasible. These elements are present in artificial sweat at concentrations several orders of magnitude higher than the fortification levels, far beyond the range typically used for trace-level validation. Under such conditions, recovery or bias calculations become analytically meaningless, as small relative variations in signal are masked by the dominant matrix background. This limitation is well recognized in ICP-MS methodology for major, easily ionizable elements in saline matrices and is addressed in analytical validation guidelines, which recommend excluding such elements from conventional recovery-based accuracy assessments when endogenous concentrations greatly exceed the added amounts.

The positive bias observed for Fe in the certified reference material may reflect residual matrix-related effects and/or incomplete correction of polyatomic interferences commonly associated with Fe determination by ICP-MS in saline matrices. Nevertheless, spike recoveries obtained at medium-to-high fortification levels remained within acceptable ranges, supporting the suitability of Fe data for exposure-oriented assessment. Similarly, although Mo and Se exhibited bias values slightly above 20% in the CRM analysis, their recoveries and repeatability under matrix-matched conditions remained satisfactory at concentration levels relevant to the analyzed samples, indicating that the matrix-matched calibration strategy substantially improved analytical performance despite minor residual matrix effects.

Overall, the results indicate that the proposed method provides reliable quantitative data for most trace elements under the adopted experimental conditions, whereas results obtained near the LOQ or strongly affected by matrix background should be interpreted with caution.

### 2.2. Leachable Elemental Concentrations

**Figure 1:**
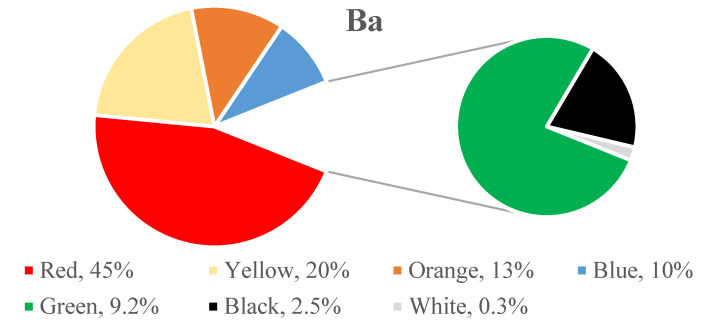

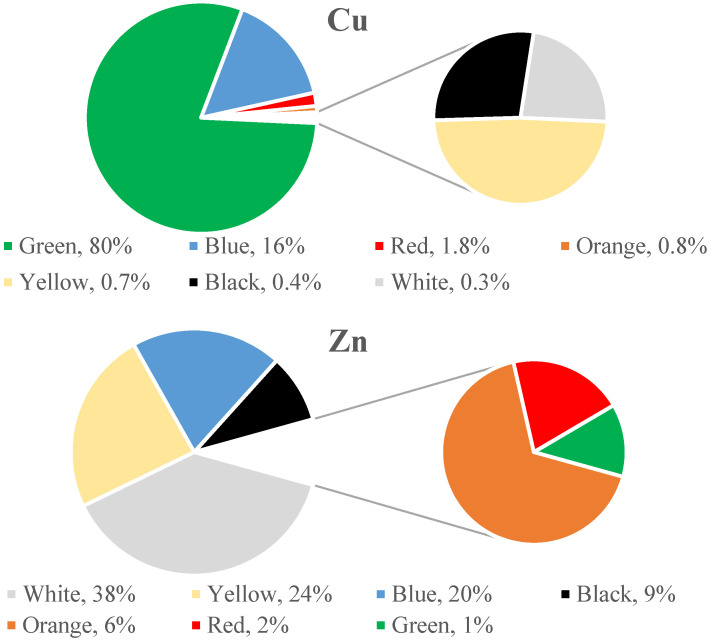
Distribution of regulated soluble Cu, Zn, and Ba in commercial tattoo inks grouped by color category.

The use of artificial sweat aimed to simulate dermal exposure conditions and to estimate the bioaccessible fraction of elements potentially released from tattoo inks. Elemental analysis of the soluble fraction revealed a highly heterogeneous distribution across colors (Table S1; Figure 1), reflecting the influence of pigment chemistry and formulation on element mobilization. Most elements were present at low concentrations, frequently near or below LODs, indicating limited bioaccessibility under the applied extraction conditions. This observation is toxicologically relevant, as the soluble fraction is more directly associated with the portion potentially available for dermal uptake and local biological interaction than total elemental content alone. In the context of tattoo safety assessment, bioaccessibility represents an important parameter for estimating realistic exposure conditions and supporting risk evaluation under the current European REACH framework. Among the more abundant elements, Ca and Mg were consistently measurable across colors, with median values ranging from 34–574 mg/kg for Ca and 41–98 mg/kg for Mg. Iron showed moderate levels (median 0.95–1.95 mg/kg) with no significant color-dependent differences. Potassium was consistently below the LOD (<30 mg/kg) in all samples. Boron displayed low but detectable levels, with slightly higher medians in yellow and green inks.

**Figure 2:**
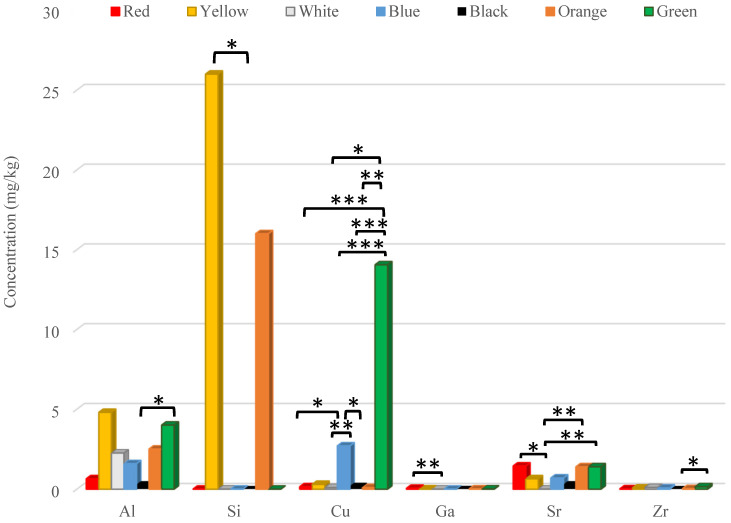
Color-dependent differences in soluble elemental concentrations in commercial tattoo inks. Significant pairwise differences are indicated as follows: Al, green > black (*); Cu, green > red, white, and black (***), green > orange (**), green > yellow (*), and blue > white (**), black (*), and red (*); Ga, red > white (**); Si, yellow > black (*); Sr, red > white (*), orange > white (**), and green > white (**); Zr, green > black (*). * *p* < 0.05; ** *p* < 0.01; *** *p* < 0.001.

Among trace elements (Figure 2), statistically significant color-dependent differences were observed for Al and Ga (*p* < 0.05 and *p* < 0.01, respectively), with higher median concentrations in yellow and green inks, likely associated with inorganic pigments or fillers. Similarly, Si and Zr showed color-dependent trends (*p* < 0.05), consistent with the use of silicate-based or zirconium-containing compounds. Detailed statistical results, including Kruskal–Wallis test statistics (H, degrees of freedom, and global *p*-values) together with Bonferroni-adjusted pairwise comparison *p*-values, were added to Table S2.

Strontium concentrations showed significant differences among ink colors (*p* < 0.05), with higher median values observed in red, orange and green samples, and the lowest levels in white inks.

A more pronounced color effect was evident for Cu, with the highest soluble fractions in green and blue inks, reaching up to 135 mg/kg in green samples. This reflects the use of Cu phthalocyanine pigments in these colors [6,29]. The strong coordination of Cu within the phthalocyanine macrocycle limits its release, so only a fraction is bioaccessible, highlighting the importance of distinguishing between total and soluble Cu for exposure and toxicological assessments [26]. Copper is an essential trace element involved in redox enzymatic processes, with tight intracellular regulation; both deficiency and excess can have adverse effects [30,31,32,33]. Excessive Cu exposure has been associated with oxidative stress and inflammatory responses due to the redox activity of Cu ions [33]. In tattooed skin, where pigments may persist for prolonged periods within dermal tissue, evaluation of the soluble Cu fraction is therefore relevant for estimating the potential availability of biologically accessible Cu species. Regulatory limits refer specifically to the soluble fraction, defined in the European Council Resolution ResAP (2008)1 [13] as the portion extracted into aqueous solution at pH 5.5, reflecting skin-relevant availability.

Zinc exhibited the highest soluble fractions in white inks, followed by yellow, blue, and black, consistent with ZnO as a white pigment and opacity modifier. Partial dissolution is promoted by ZnO’s amphoteric behavior and surface reactivity, influenced by pH, particle characteristics, and matrix interactions [34,35,36]. Although Zn is an essential element, elevated concentrations of soluble Zn species may contribute to local cytotoxicity and inflammatory effects under certain exposure conditions. The measurable release observed in this study confirms that ZnO-containing pigments may represent a relevant source of bioaccessible Zn in tattoo formulations.

Barium was mainly detected in yellow, green, and blue inks, reflecting its use as BaCrO_4_ in bright yellow pigments and BaSO_4_ to stabilize organic pigments [18,23,24]. The measurable soluble fraction suggests the presence of more labile species or matrix-mediated mobilization, which is relevant from a regulatory perspective, as limits apply specifically to soluble Ba [15,24]. From a toxicological perspective, soluble Ba compounds are of greater concern than insoluble forms because they can more readily interact with biological systems and contribute to systemic exposure. The detection of soluble Ba, even below current regulatory thresholds, therefore supports the relevance of extraction-based approaches for exposure-oriented risk assessment. Although red inks showed higher median Ba values, differences across colors were not statistically significant, highlighting the variability among formulations and the need for cautious interpretation, particularly given the limited comparability with literature data obtained under different extraction conditions.

Elements regulated for total content—including Ni, Pb, Sb, Sn, Cd, and Co—were generally present at low concentrations in the soluble fraction, without significant color effects. As, Be, Se, and several rare earth elements (Nd, Pr, Tb) were below LODs, indicating negligible mobilization. The limited release observed for these toxicologically relevant elements suggests low bioaccessibility under the adopted extraction conditions, although this does not exclude possible long-term accumulation or release under more complex in vivo conditions. Major elements such as Ca, Mg, and K reflected the ink matrix or additives rather than pigments.

Soluble Ba, Cu, and Zn were consistently below the regulatory limits of Commission Regulation (EU) 2020/2081 [14] (Ba: 500 mg/kg; Cu: 250 mg/kg; Zn: 2000 mg/kg; Table S1). The pronounced color-dependent patterns observed for these elements (Figure 1) confirm that bioaccessibility is primarily governed by pigment chemistry and matrix interactions, which is crucial for regulatory compliance and risk evaluation. These findings support the toxicological relevance of assessing soluble elemental fractions in addition to total concentrations, particularly because current European REACH restrictions specifically address the bioavailable fraction considered potentially accessible under skin-relevant conditions.

It is important to interpret these results with caution. The use of artificial sweat provides a simplified model of dermal exposure and may not fully replicate complex in vivo conditions, such as prolonged retention in skin tissue, local pH variations, or interactions with proteins and lipids. Furthermore, tattoo pigments may undergo long-term physicochemical transformations within the dermis, potentially altering metal release kinetics and bioavailability over time. Therefore, the present extraction approach should be considered an exposure-oriented screening tool rather than a direct predictor of clinical outcomes or systemic toxicity. Additionally, comparison with literature data is challenging due to substantial methodological variability, including differences in extraction protocols, digestion procedures, analytical techniques, and ink formulations [6,15]. As summarized in Table S3, most studies published over the past decade rely on total acid digestion (commonly using HNO_3_, often combined with HCl, H_2_O_2_, and/or HF) followed by ICP-based techniques (ICP-MS or ICP-OES) for multi-elemental quantification [17,18,19,37,38,39]. However, some approaches also include the assessment of soluble fractions (e.g., NaCl extraction) or direct dilution strategies [25,40], leading to differences in the measured metal concentrations. Furthermore, the number of elements analyzed and the sample preparation protocols vary considerably across studies, ranging from targeted analysis of a few toxic metals (e.g., Cd, Pb, Hg) to comprehensive multi-element screening. This heterogeneity limits direct compara-bility and highlights the need for harmonized analytical methodologies.

These findings further support the need for validated extraction protocols that more realistically reflect dermal exposure scenarios. While most elements exhibit limited solubility and a consequently low potential for skin uptake, measurable soluble fractions of Ba, Cu, and Zn highlight the importance of formulation-specific assessments, as metal release is strongly influenced by ink composition and extraction conditions. This underscores the relevance of such evaluations for both toxicological interpretation and regulatory risk assessment.

## 3. Materials and Methods

### 3.1. Chemicals and Materials

All reagents were of analytical grade and of high purity. Deionized water (resistivity 18.2 MΩ cm) produced using a water purification system (Arioso Power I ROUP Scholar UV; Human Corporation, Songpa-Ku, Seoul, Korea) was used for preparing solutions, blanks, and calibration standards. Artificial sweat (purity 99.9%; pH ≈ 5.5; Biochemazone Inc., Leduc, AB, Canada) was used to extract the tattoo ink samples. The certified reference material NIST 1643f (Trace Elements in Water, Exaxol Italia Chemical Manufacturer S.R.L., Genova, Italy) was used to verify the accuracy of the analytical procedure. Throughout the study, graduated polypropylene tubes (2.5–10 mL; Artiglass S.R.L., Due Carrare, Italy), single-use syringes (Injekt, B. Braun Melsungen AG, Melsungen, Germany), and adjustable micropipettes (Gilson Inc., Middleton, WI, USA) fitted with disposable tips (EPT I.P.S. Standard Bulks, Hamburg, Germany) were employed. Prior to instrumental measurements, samples were filtered using syringe-mounted membrane filters, either mixed cellulose ester (0.45 µm; Exacta + Optech Labcenter S.p.A., Modena, Italy) or cellulose acetate (0.20 µm; Merck KGaA, Darmstadt, Germany).

For ICP-MS analysis, multi-element custom standard solutions (CPAChem, Bogomilovo, Bulgaria) were used for external calibration. Internal standard solutions of Y (0.005 mg/L, prepared from 1000 ± 2 mg/L stock solutions; Panreac Química, Barcelona, Spain) and In, Sc, Th, and Rh (0.010 mg/L, from 1000 ± 5 mg/L stocks; Merck KGaA, Darmstadt, Germany) were prepared in 2% (*v*/*v*) HNO_3_ (Suprapure grade; Carlo Erba Reagents, Milan, Italy). Instrument performance was monitored daily using a multi-element tuning solution containing Ba, Be, Ce, Co, In, Pb, Mg, Tl, and Th (0.005 mg/L, prepared from a 10.00 ± 0.05 mg/L stock; Spectro Pure, Ricca Chemical Company, Arlington, TX, USA). This solution was employed to optimize sensitivity, limit oxide and doubly charged ion formation, and perform mass calibration prior to analysis.

### 3.2. Instrumentation

All ink samples were weighed using an Analytical balance Europe 60 (Gibertini Elettronica, Milan, Italy) with a readability of 0.1 mg. Sample extractions were carried out in a WB12 thermostated water bath (Argo Lab, Modena, Italy) equipped with electronic temperature control. Elemental determinations were performed by quadrupole ICP-MS using an 820-MS instrument (Bruker, Bremen, Germany) fitted with a collision–reaction interface (CRI). High-purity He and H_2_ gases (99.9995%; SOL Spa, Monza, Italy) were employed to reduce isobaric and polyatomic interferences. The CRI operated in no-gas mode for most analytes, whereas As, Ca, Cr, Fe, Mn, Se, and V were measured using H_2_ (70 mL min^−1^) as the skimmer gas and He (30 mL min^−1^) as the sampler gas. Data acquisition was carried out in steady-state mode with peak-hopping scanning and fine mass spacing. Each isotope was measured using one point per peak, five scans per replicate, and three replicates per sample. Detailed instrumental operating conditions are reported in Table 1.

### 3.3. Preparation of Tattoo Ink Samples

78 tattoo inks from eight brands, covering seven colors and their different shades (yellow: n = 10, green: n = 12, blue: n = 14, red: n = 13, orange: n = 7, white: n = 9, and black: n = 13), were purchased from various sellers on Amazon in 2022 at relatively low cost. The products originated from China, the United States, and Austria and included tattoo and microblading inks with different formulations (permanent, semi-permanent, plant-based, vegan, and products marketed as non-toxic or organic). All inks were supplied in sealed containers (5–60 mL), homogenized before analysis, and stored at room temperature in the dark.

According to label information, the formulations mainly consisted of water, alcohols, and polyols (e.g., ethanol, isopropanol, propylene glycol, glycerin/glycerol), witch hazel extract, and pigments, with some products also containing preservatives or stabilizers. Overall, the declared compositions are consistent with conventional tattoo ink formulations designed to ensure pigment dispersion, dermal delivery, and product stability [8].

The leachable elemental fraction of the tattoo inks was evaluated using artificial sweat as the extraction medium. For each sample, approximately 250 mg of ink were combined with 5.0 mL of extraction solution and incubated at 37 °C for 1 h in a thermostated water bath, as suggested by Prior (2015) [15]. This procedure was adapted from Wang et al. (2021) [25] with a shorter extraction time to focus on initial metal release under physiologically relevant conditions. Since the European Council Resolution ResAP(2008)1 [13] specifies extraction of soluble copper at pH 5.5 but does not define an extraction duration. After incubation, samples were sequentially filtered through 0.45 µm and 0.20 µm syringe filters to remove suspended particles. The extracts were diluted 1:10 with 2% (*v*/*v*) HNO_3_ prior to ICP-MS analysis to lower TDS, reducing matrix-induced signal suppression and preventing salt deposition in the sample introduction system, in accordance with instrument guidelines and standard analytical practices.

### 3.4. Quality Control and Accuracy Assessment

Method performance was assessed by evaluating precision, accuracy, linearity as well as LOD, LOQ, and instrumental drift.

For each element (Al, As, B, Ba, Be, Bi, Ca, Cd, Ce, Co, Cr, Cs, Cu, Dy, Fe, Ga, Gd, K, La, Li, Mg, Mn, Mo, Na, Nb, Nd, Ni, P, Pb, Pr, Rb, Sb, Se, Si, Sn, Sr, Tb, Te, Ti, Tl, U, V, W, Zn, and Zr), calibration standards were prepared in matrices matching those of the extracted samples (artificial sweat diluted 1:10) to ensure matrix-consistent quantification. For ICP-MS, at least three-point calibration curves were constructed (Table 2), linearity was verified using the instrument software, and a correlation coefficient (R^2^) ≥ 0.999 was required for acceptance.

Limits of detection and quantification (Table 2) were determined as three and ten times, respectively, the standard deviation of repeated blank measurements (n = 10).

Method accuracy (bias% or recovery%) and intra-day precision (RSD %) were assessed through the analysis of the certified reference material NIST 1643f and spiked samples. Four spiking levels were applied. Level 1 concentrations were: 0.5 µg/L for Al, As, Ba, Be, Bi, Cd, Ce, Co, Cr, Cs, Cu, Dy, Ga, Gd, La, Li, Mn, Mo, Nb, Nd, Ni, Pb, Pr, Rb, Sb, Se, Sn, Tb, Te, Ti, Tl, U, V, W and Zr; 5 µg/L for Fe and Zn; 25 µg/L for P and Si; 27.5 µg/L for B and Sr; 250 µg/L for Na, K and Mg; and 500 µg/L for Ca. Levels 2, 3 and 4 correspond approximately to 2×, 10× and 40× the Level 1 concentrations, respectively (Table 3). Inter-sample repeatability was also evaluated as a fundamental parameter to assess potential differences in element concentrations among different sample types, according to the following expressions (1), in accordance with Astolfi et al. (2017) [41]:

(1) $$r_{rel}=\Delta /\bar{x}\times 100;\Delta =\sqrt{\frac{\sum_{i=1}^{N}{\left(C_{i}B-C_{i}A\right)}^{2}}{2N}};\;\bar{x}=\;\frac{\sum_{i=1}^{N}(C_{i}B+C_{i}A)}{2N}$$

Instrumental stability was monitored by analyzing a fifth calibration standard immediately after calibration, at 20-sample intervals, and at the end of each analytical batch. A maximum acceptable drift of ±10% was applied; when exceeded, corrective actions such as instrument re-recalibration were undertaken, and the preceding 20 samples were reanalyzed.

Finally, potential matrix-related effects and signal fluctuations due to temperature variations, cone fouling, or other instrumental instabilities during ICP-MS analysis were compensated using a set of internal standards (Sc, Y, Rh, In, and Th), which were added to all blanks, calibration standards, and samples.

### 3.5. Data Elaboration

Data processing and statistical analyses were carried out using IBM SPSS Statistics 27 (IBM Corp., Armonk, NY, USA) and Microsoft Excel (Microsoft Corp., Redmond, WA, USA). The normality of data distribution was assessed using the Kolmogorov–Smirnov and Shapiro–Wilk tests. As normality assumptions were not met, non-parametric statistical analysis was applied. Differences in elemental concentrations among tattoo ink color groups were evaluated using the Kruskal–Wallis test for independent samples, followed by pairwise post hoc comparisons to identify statistically significant differences between specific color categories. Statistical significance was set at *p* < 0.05.

Elements for which ≥50% of the measured values were below the LOD were excluded from statistical analysis, namely As, Be, Bi, Cr, Dy, Gd, K, La, Nb, Nd, P, Pb, Pr, Rb, Se, Tb, Te, Tl, and V. Sodium was also excluded due to its high background levels associated with the artificial sweat matrix.

## 4. Conclusions

This study evaluated the leachable elemental fraction of commercial tattoo inks using artificial sweat extraction coupled with ICP-MS analysis, providing an exposure-oriented assessment of elements potentially available for dermal uptake under sweat-simulated conditions. The proposed extraction protocol enabled the characterization of the bioaccessible fraction of a broad range of elements while minimizing excessive sample treatment associated with total digestion procedures.

The results demonstrated pronounced color-dependent differences in elemental release, reflecting the influence of pigment chemistry and formulation. In particular, green and blue inks showed the highest soluble Cu concentrations, whereas white and yellow inks exhibited higher soluble Zn levels. Detectable soluble Ba was mainly observed in yellow, green, and blue inks, suggesting the presence of Ba-containing pigments or fillers. Additional statistically significant differences were observed for Al, Ga, Si, Sr, and Zr, indicating formulation-specific variability in elemental mobility. Despite these differences, soluble Ba, Cu, and Zn concentrations remained below the limits established by Commission Regulation (EU) 2020/2081 [14].

Most regulated toxic elements, including Cd, Co, Ni, Pb, Sb, and Sn, were present at low concentrations or below detection limits in the soluble fraction, indicating limited mobilization under the applied extraction conditions. These findings highlight that total elemental content alone may overestimate actual exposure potential, as only a fraction of the elements incorporated in pigments and additives appears bioaccessible under mild physiological conditions.

Artificial sweat introduced substantial matrix-related interferences due to its high salt and chloride content. However, the combined use of sample dilution, internal standard correction, CRC technology, and matrix-matched calibration effectively mitigated spectral and non-spectral effects, ensuring reliable quantification. Method validation confirmed satisfactory accuracy, precision, and reproducibility for trace element determination.

Overall, the proposed methodology provides a robust and transferable framework for investigating the bioaccessible elemental fraction of tattoo inks under realistic exposure-related conditions. The observed formulation- and color-dependent release patterns emphasize the importance of harmonized extraction procedures for toxicological evaluation, regulatory compliance assessment, and future risk characterization of tattoo inks.

## Figures and Tables

**Table 1:** ICP-MS operation parameters.

Uptake time	50 s
Stabilization time	45 s
Rinse time	120 s
Monitored mass	Li7, Be9, B11, Na23, Mg24, Al27, Si28, P31, K39, Ti49, Co59, Ni60, Cu65, Zn66, Ga69, Rb85, Sr88, Zr90, Nb93, Mo98, Cd112, Sn118, Sb121, Te125, Cs133, Ba137, La139, Ce140, Pr141, Nd143, Gd157, Tb159, Dy163, W182, Tl205, Pb208, Bi209, U238, Ca44, V51, Cr52, Mn55, Fe56, As75, Se76
Internal standards	Sc45, Y89, Rh103, In115, Th232
RF power	1400 W
Plasma Ar flow	18.0 L/min
Auxiliary Ar flow	1.80 L/min
Nebulizer Ar flow	0.90 L/min
Sheath Ar flow	0.16 L/min
Pump speed	3 rpm
Collision cell gas	H_2_, He
Cell H_2_ flow	70 L/min
Cell He flow	30 L/min

**Table 2:** Comparison of calibration parameters obtained in 2% HNO_3_ and artificial sweat solution.

Solvent (2% HNO_3_)												Matrix (Artificial Sweat)					
Element	n ^a^	Slope ^b^	Intercept ^b^	Calibration Curve Range ^c^	R^2 d^	BEC ^e^	LOD ^f^	LOQ ^f^	n ^a^	Slope ^b^	Intercept ^b^	Calibration Curve Range ^c^	R^2 d^	BEC ^e^	LOD ^f^	LOQ ^f^	Δ% ^g^
Al	7	40,670	435,674	0–50	0.9876	11	0.1	0.2	7	25,590	225,472	0–50	0.9985	9	0.1	0.2	−37
As	6	161	33	0–10	0.9996	0.2	0.01	0.02	4	48	90	0–10	0.9935	0.5	0.1	0.3	−70
B	4	14,290	74,326	0–55	>0.9999	5	0.04	0.1	4	5702	55,793	0–55	0.9999	10	0.1	0.5	−60
Ba	9	8504	16,580	0–50	0.9977	2	0.03	0.09	6	7673	14,155	0–50	0.9993	2	0.03	0.1	−10
Be	5	20,390	30.7	0–2	>0.9999	0.002	0.00003	0.0001	5	11,520	33	0–2	0.9999	0.003	0.001	0.002	−44
Bi	5	57,000	122	0–5	>0.9999	0.002	0.0002	0.0005	5	51,160	97	0–5	0.9999	0.002	0.0004	0.001	−10
Ca	6	98.5	2857	0–10,000	0.9998	30	7	20	4	62.9	19,044	0–10,000	0.9995	300	8	30	−36
Cd	6	12,630	22	0–5	0.9999	0.002	0.00003	0.0001	5	7968	41	0–2	0.9994	0.005	0.001	0.003	−37
Ce	4	89,850	919	0–1	0.9998	0.01	0.0003	0.001	4	66,100	433	0–1	>0.9999	0.007	0.0003	0.001	−26
Co	5	42,170	685	0–2	>0.9999	0.02	0.0002	0.0006	4	27,410	568	0–5	0.9998	0.02	0.002	0.006	−35
Cr	5	595	79	0–20	0.9989	0.1	0.03	0.1	4	314	873	0–10	0.9996	2	0.4	1	−47
Cs	4	89,980	105	0–1	>0.9999	0.001	0.00003	0.0001	4	58,460	140	0–1	0.9999	0.002	0.0003	0.001	−35
Cu	8	8808	1338	0–50	>0.9999	0.2	0.01	0.02	8	4731	2971	0–50	0.9996	0.6	0.003	0.01	−46
Dy	4	20,790	151	0–1	0.9990	0.0006	0.00004	0.0001	4	17,580	47	0–1	0.9996	0.003	0.0004	0.001	−15
Fe	8	3877	1269	0–500	0.9923	0.3	0.3	1	8	2006	2777	0–500	0.9997	1	0.3	1	−48
Ga	4	37,340	1770	0–1	>0.9999	0.05	0.001	0.003	4	24,270	1487	0–1	0.9999	0.06	0.004	0.01	−35
Gd	4	17,730	−42	0–1	0.9999	0.0008	0.0001	0.0002	4	14,420	13	0–1	>0.9999	0.0009	0.001	0.003	−19
K	5	30,340	9,289,331	0–1000	0.9985	310	1	3	3	13,790	239,817,808	0–25,000	0.9995	16,400	30	110	−55
La	4	94,540	625	0–1	0.9999	0.007	0.0002	0.0008	4	65,180	363	0–1	>0.9999	0.006	0.001	0.002	−31
Li	6	92,580	1479	0–5	>0.9999	0.02	0.0002	0.0005	5	44,580	2093	0–2	0.9999	0.05	0.001	0.003	−52
Mg	6	20,520	51,670	0–5000	>0.9999	3	0.4	1	5	11,730	170,926	0–5000	0.9999	14	1	2	−43
Mn	7	5119	793	0–20	>0.9999	0.2	0.01	0.02	8	2686	317	0–50	0.9998	0.1	0.1	0.2	−48
Mo	9	15,200	178	0–50	0.9968	0.01	0.009	0.03	9	10,840	1071	0–50	0.9995	0.1	0.006	0.02	−29
Na	6	28,200	682,656	0–25,000	0.9966	24	30	90	4	nd ^h^	nd	*Signal not increasing*	nd ^h^	nd ^h^	nd ^h^	nd ^h^	nd ^h^
Nb	5	70,840	42.7	0–2	>0.9999	0.0006	0.0001	0.0003	5	46,290	368	0–2	0.9998	0.008	0.0007	0.002	−35
Nd	4	14,590	−16.4	0–1	0.9997	0.003	0.0002	0.0008	4	16,220	100	0–1	0.9998	0.006	0.0004	0.0012	11
Ni	8	8234	1616	0–20	0.9992	0.2	0.02	0.07	6	5051	1076	0–10	0.9997	0.2	0.008	0.03	−39
P	3	898	132,565	0–2500	0.9947	150	8	30	3	480	2,645,907	0–5000	0.9993	5500	280	920	−47
Pb	5	35,590	1140	0–2	>0.9999	0.03	0.001	0.005	5	31,940	2273	0–2	0.9999	0.07	0.01	0.05	−10
Pr	4	132,200	−339	0–1	0.9999	0.001	0.00003	0.0001	4	95,370	80	0–1	0.9999	0.0008	0.00003	0.0001	−28
Rb	4	60,270	1026	0–5	0.9999	0.02	0.001	0.002	5	36,410	19,248	0–5	>0.9999	0.5	0.02	0.06	−40
Sb	7	23,320	40	0–10	0.9999	0.002	0.005	0.02	6	12,100	869	0–5	0.9998	0.07	0.003	0.009	−48
Se	6	73	34	0–10	0.9988	0.5	0.007	0.02	4	72	33	0–2	0.9930	0.5	0.1	0.3	−1
Si	5	8253	3,417,851	0–2500	0.9947	410	1	3	4	6349	5,203,768	0–2500	0.9979	820	10	40	−23
Sn	4	19,430	113	0–1	>0.9999	0.006	0.0002	0.0005	4	13,150	139	0–1	0.9999	0.01	0.0003	0.001	−32
Sr	4	78,960	36,381	0–55	>0.9999	0.5	0.02	0.06	3	54,200	116,268	0–55	>0.9999	2	0.1	0.4	−31
Tb	4	114,900	−1130	0–1	0.9995	0.0001	0.00001	0.00002	4	95,080	7	0–1	0.9997	0.0001	0.0001	0.0003	−17
Te	4	1206	10.7	0–1	0.9999	0.009	0.001	0.002	4	907	16	0–1	0.9998	0.02	0.007	0.025	−25
Ti	9	3383	1060	0–50	0.9993	0.3	0.002	0.006	7	1878	2621	0–20	0.9985	1	0.04	0.1	−44
Tl	4	47,380	37	0–2	>0.9999	0.0008	0.000003	0.000012	4	46,680	92	0–2	0.9999	0.002	0.001	0.002	−1
U	6	87,540	55	0–5	>0.9999	0.0006	0.00003	0.0001	6	57,010	80	0–5	0.9999	0.001	0.0001	0.0004	−35
V	4	1789	92	0–10	0.9993	0.05	0.02	0.08	4	1032	574	0–10	0.9999	0.6	0.2	1	−42
W	6	17,530	169	0–5	>0.9999	0.01	0.001	0.003	9	14,300	339	0–50	0.9994	0.02	0.003	0.01	−18
Zn	8	4785	13,722	0–500	0.9939	3	0.2	0.8	6	3288	4473	0–200	0.9973	1	0.3	0.9	−31
Zr	8	43,510	144	0–20	0.9998	0.003	0.0001	0.0002	7	28,010	1192	0–10	0.9992	0.04	0.001	0.004	−36

^a^ n, number of concentration levels including blank. ^b^ Calibration Equation: cps = (a conc + b) [I/S Ratio], where: a, slope; b, intercept; I/S = Analyte intensity (cps)/Internal standard intensity (cps); (cps = counts per second). ^c^ Calibration curve range in μg/L. ^d^ Correlation coefficient. ^e^ BEC: background equivalent concentration (μg/L). ^f^ LOD: limit of detection, LOQ: limit of quantification (mg/kg). ^g^ Δ% percentage bias, calculated as the relative difference between matrix-matched and aqueous calibration slopes. ^h^ nd: not determinable.

**Table 3:** Method accuracy (bias, %) and precision (RSD, %) evaluated using the certified reference material NIST 1643f and spike recoveries in fortified samples at four concentration levels; repeatability (rrel, %) was also assessed on pairs of real samples.

	NIST 1643f ^a^			Spike—Level 1 ^b^		Spike—Level 2 ^b^		Spike—Level 3 ^b^		Spike—Level 4 ^b^		
Element	Certified Value	Bias%	RSD%	R%	RDS%	R%	RDS%	R%	RDS%	R%	RDS%	r_rel_^c^
Al	133.8	11	7	>120	10	>120	11	>120	7	117	0.1	14
As	57.42	9	2	>120	25	>120	17	115	8	120	4	nd
B	152.3	−3	2	120	6	104	2	93	0.1	117	0.1	14
Ba	518.2	−3	2	>120	16	99	13	115	3	108	1	1
Be	13.17	−5	1	85	2	74	1	84	1	96	0.2	nd
Bi	12.62	11	4	91	2	76	2	85	5	94	3	nd
Ca	29,430	−6	3	93	7	91	1	78	1	86	2	6
Cd	5.89	15	12	90	2	79	0.3	87	0.03	102	1	18
Ce	-	nd	nd	88	1	78	1	86	3	100	1	20
Co	25.3	−4	2	85	7	72	4	83	2	92	2	10
Cr	18.5	−10	12	>120	20	120	20	80	2	80	12	nd
Cs	-	nd	nd	95	3	83	3	92	2	104	2	13
Cu	21.66	12	3	>120	8	119	5	96	2	99	0.2	10
Dy	-	nd	nd	85	4	83	5	91	1	103	5	nd
Fe	93.44	20	9	81	14	119	4	78	0.03	80	5	15
Ga	-	nd	nd	97	1	83	1	96	1	99	0.4	6
Gd	-	nd	nd	87	12	76	6	78	6	99	2	nd
K	1932.6	nd	nd	>120	1	>120	1	>120	4	108	0.1	nd
La	-	nd	nd	92	1	84	1	94	2	104	0.1	nd
Li	16.59	6	2	97	1	81	0.2	89	1	100	1	18
Mg	7454	1	3	84	6	74	5	85	1	101	0.4	4
Mn	37.14	2	13	82	56	87	20	94	5	90	1	4
Mo	115.3	23	6	>120	1	114	1	109	8	120	1	7
Na	18,830	nd	nd	>120	2	>120	1	>120	1	>120	0.5	nd
Nb	-	nd	nd	102	3	87	1	101	2	106	1	nd
Nd	-	nd	nd	88	7	90	7	99	12	106	2	nd
Ni	59.8	−3	1	107	8	88	2	87	2	92	6	17
P	-	nd	nd	>120	3	>120	6	>120	3	109	1	nd
Pb	18.488	9	11	102	2	80	0.4	86	4	96	2	nd
Pr	-	nd	nd	98	2	85	2	93	8	110	0.4	nd
Rb	12.64	22	5	>120	1	>120	2	105	2	100	0.5	nd
Sb	55.45	11	0	109	0.2	91	1	94	1	108	2	12
Se	11.7	21	8	>120	27	118	15	120	2	118	2	nd
Si	-	nd	nd	>120	2	>120	4	>120	5	120	6	12
Sn	-	nd	nd	100	2	88	1	93	1	110	2	11
Sr	314	18	2	80	1	91	0	80	2	120	3	0.2
Tb	-	nd	nd	99	3	87	3	94	4	108	1	nd
Te	0.977	22	7	118	11	105	8	108	1	120	1	nd
Ti	-	nd	nd	>120	2	>120	1	108	2	106	2	11
Tl	6.892	−5	3	84	9	71	2	82	2	87	5	15
U	-	nd	nd	91	0	81	4	90	3	98	1	13
V	36.07	−9	24	>120	40	>120	80	119	10	89	2	nd
W	-	nd	nd	118	1	98	0.3	102	4	113	3	0.7
Zn	74.4	−5	3	>120	3	119	6	86	0.4	87	2	4
Zr	-	nd	nd	101	0	91	1	99	1	102	1	10

^a^ The symbol “–” indicates that no certified value is available for that chemical element in NIST 1643f; nd indicates that the value is not determinable. ^b^ Four spiking levels were applied. Level 1 concentrations (µg/L) were: 0.5 for Al, As, Ba, Be, Bi, Cd, Ce, Co, Cr, Cs, Cu, Dy, Ga, Gd, La, Li, Mn, Mo, Nb, Nd, Ni, Pb, Pr, Rb, Sb, Se, Sn, Tb, Te, Ti, Tl, U, V, W and Zr; 5 for Fe and Zn; 25 for P and Si; 27.5 for B and Sr; 250 for Na, K and Mg; and 500 for Ca. Levels 2, 3 and 4 correspond approximately to 2×, 10× and 40× the Level 1 concentration, respectively. ^c^ nd: not determinable; repeatability on pairs of real samples could not be calculated because more than 50% of the samples were below the limit of detection (LOD).

## Data Availability

The data presented in this study are available on request from the corresponding author.

## Supplementary Materials

The following supporting information can be downloaded at: https://www.mdpi.com/article/10.3390/molecules31111804/s1, Table S1a,b: Extractable elemental concentrations (mg/kg) in tattoo inks using artificial sweat. Table S2: Kruskal–Wallis test statistics and Bonferroni-adjusted pairwise comparisons for elements showing significant color-dependent differences in soluble elemental concentrations in commercial tattoo inks. Table S3: Summary of studies published in the last 10 years on the elemental composition of tattoo inks.

## Abbreviations

The following abbreviations are used in this manuscript:

BEC	Background equivalent concentration
CRC	Collision/reaction cell
FAAS	Flame atomic absorption spectrometry
GF-AAS	Graphite furnace atomic absorption spectrometry
ICP-OES	Inductively coupled plasma optical emission spectrometry
ICP-MS	Inductively coupled plasma mass spectrometry
ISC	Internal standard correction
LOD	Limit of detection
LOQ	Limit of quantification
REACH	Registration, Evaluation, Authorization and Restriction of Chemicals
RSD%	Relative standard deviation percentage
SP-ICP-MS	Single particle inductively coupled plasma mass spectrometry
TDS	Total dissolved solids
