# Supplementary material for: Assessment of Dermally Bioaccessible Elements by Sweat-Simulated Extraction: Analytical Approach and Application to Tattoo Inks
Source: Molecules. 2026 May 24;31(11):1804. doi: 10.3390/molecules31111804 (PMC13257482; doi:10.3390/molecules31111804)
Supplement: Supplementary file 1 [file molecules-31-01804-s001.zip › molecules-4308563-supplementary.pdf]

Supplementary material

Assessment of dermally bioaccessible elements by sweat-simulated extraction: analytical approach and application to tattoo inks

Carmela Protano [1], Arianna Antonucci [1] and Maria Luisa Astolfi [2,*]

[1] Department of Benessere, Salute e Sostenibilità Ambientale, Sapienza University of Rome,
00185 Rome, Italy; carmela.protano@uniroma1.it (C.P.); arianna.antonucci@uniroma1.it (A.A.)
2 Department of Chemistry, Sapienza University of Rome, 00185 Rome, Italy
* Correspondence: marialuisa.astolfi@uniroma1.it; Tel.: +39-0649913748

Table S1a. Extractable elemental concentrations (mg/kg) in tattoo inks using artificial sweat.

	Red (n = 13)			Yellow (n = 10)			White (n = 9)			Blue (n = 14)			Black (n =13)			Orange (n = 7)			Green (n = 12)			
Element	median	min	max	median	min	max	median	min	max	median	min	max	median	min	max	median	min	max	median	min	max	p
Al	0.65	<0.1	6.1	4.8	<0.1	10.8	2.25	<0.1	19.9	1.6	<0.1	18.7	**0.3**^{a}	<0.1	1.2	2.5	<0.1	9.5	**4.0**^{a}	0.4	13.7	**<0.05**
As	<0.1	<0.1	<0.1	<0.1	<0.1	<0.1	<0.1	<0.1	<0.1	<0.1	<0.1	<0.1	<0.1	<0.1	<0.1	<0.1	<0.1	<0.1	<0.1	<0.1	<0.1	nd
B	0.3	<0.1	1	0.6	<0.1	3.2	<0.1	<0.1	1.6	0.3	<0.1	1.6	0.55	<0.1	0.80	0.5	<0.1	1.8	0.135	<0.1	2.1	ns
Ba	1.81	0.12	9.28	0.21	<0.03	7.39	<0.03	<0.03	<0.03	0.10	<0.03	3.78	0.05	<0.03	0.96	0.53	0.09	3.44	0.38	0.17	2.25	ns
Be	<0.001	<0.001	<0.001	<0.001	<0.001	<0.001	<0.001	<0.001	<0.001	<0.001	<0.001	<0.001	<0.001	<0.001	<0.001	<0.001	<0.001	<0.001	<0.001	<0.001	<0.001	nd
Bi	<0.0004	<0.0004	0.0182	<0.0004	<0.0004	0.0102	<0.0004	<0.0004	0.0012	<0.0004	<0.0004	0.0091	0.0012	<0.0004	0.1098	0.0004	<0.0004	0.0185	<0.0004	<0.0004	0.007	nd
Ca	141	32	555	101	67	189	34	<8	80	118	30	509	45	<8	380	358	94	609	574	41	847	ns
Cd	0.002	<0.001	0.017	0.002	0.0005	0.022	<0.001	<0.001	0.003	0.003	<0.001	0.02	0.0045	<0.001	0.040	<0.001	<0.001	0.004	<0.001	<0.001	0.018	ns
Ce	0.0005	<0.0003	0.0081	<0.0003	<0.0003	0.0107	0.0008	<0.0003	0.0030	0.0013	<0.0003	0.0407	<0.0003	<0.0003	0.0087	0.0004	<0.0003	0.0037	0.0011	<0.0003	0.0078	ns
Co	0.0045	<0.002	0.019	0.002	0.002	0.065	<0.002	<0.002	<0.002	0.005	<0.002	0.193	0.009	<0.002	0.041	0.006	<0.002	0.05	0.006	<0.002	0.021	ns
Cr	<0.4	<0.4	0.8	0.2	0.2	2.9	<0.4	<0.4	<0.4	<0.4	<0.4	0.6	<0.4	<0.4	0.5	<0.4	<0.4	2.1	<0.4	<0.4	1	nd
Cs	0.00025	<0.0003	0.0144	0.0009	<0.0003	0.0226	<0.0003	<0.0003	0.0139	0.0035	<0.0003	0.0185	0.0046	<0.0003	0.1285	0.0043	<0.0003	0.017	0.0005	<0.0003	0.0393	ns
Cu	**0.15**^{a,b}	<0.003	6.5	**0.30**^{c}	<0.003	0.60	**0.10**^{d,e}	<0.003	0.50	**2.7**^{a,d,f}	0.20	19.2	**0.20**^{f,g}	<0.003	0.40	**0.10**^{h}	0.10	1.10	**14.1**^{b,c,e,g,h}	0.70	135	**a,c,f <0.05; d,h <0.01; b,e,g <0.001**
Dy	<0.0004	<0.0004	<0.0004	<0.0004	<0.0004	<0.0004	<0.0004	<0.0004	<0.0004	<0.0004	<0.0004	<0.0004	<0.0004	<0.0004	<0.0004	<0.0004	<0.0004	<0.0004	<0.0004	<0.0004	<0.0004	nd
Fe	0.95	0.7	11.1	1.35	0.7	8.3	<0.3	<0.3	0.7	1.7	0.5	8.3	1.15	0.7	6.2	1.5	0.4	15.4	1.95	0.70	12.4	ns
Ga	**0.051**^{a}	<0.004	0.259	0.013	<0.004	0.293	**<0.004**^{a}	<0.004	<0.004	<0.004	<0.004	0.111	0.008	<0.004	0.029	0.013	<0.004	0.090	0.0125	<0.004	0.063	**<0.01**
Gd	<0.001	<0.001	<0.001	<0.001	<0.001	<0.001	<0.001	<0.001	<0.001	<0.001	<0.001	<0.001	<0.001	<0.001	<0.001	<0.001	<0.001	<0.001	<0.001	<0.001	<0.001	nd
K	<30	<30	<30	<30	<30	<30	<30	<30	<30	<30	<30	<30	<30	<30	<30	<30	<30	<30	<30	<30	<30	nd
La	<0.001	<0.001	<0.001	<0.001	<0.001	<0.001	<0.001	<0.001	<0.001	<0.001	<0.001	<0.001	<0.001	<0.001	<0.001	<0.001	<0.001	<0.001	<0.001	<0.001	<0.001	nd
Li	0.031	0.008	0.062	0.028	0.006	0.051	0.015	<0.001	0.115	0.050	0.016	0.435	0.037	0.002	1.163	0.034	0.014	0.066	0.036	0.004	0.437	ns
Mg	98	37	232	47	10	75	45	2	99	47	12	152	41	16	135	86	42	129	65	36	115	ns
Mn	0.75	<0.06	15	0.2	0.03	1.6	<0.06	<0.06	1.7	0.30	<0.06	2.9	0.2	<0.06	0.7	0.70	0.20	1.90	0.20	<0.06	0.70	ns

Non-parametric Kruskal–Wallis test for independent samples followed by pairwise comparisons was applied: "-" = not determined; "ns" = not significant at $p > 0.05$; and the data in bold with the same superscript letters within rows were significantly different ($p < 0.05$).

Table S1b. Extractable elemental concentrations (mg/kg) in tattoo inks using artificial sweat.

	**Red (n = 13)**			**Yellow (n = 10)**			**White (n = 9)**			**Blue (n = 14)**			**Black (n =13)**			**Orange (n = 7)**			**Green (n = 12)**			
Element	median	min	max	median	min	max	median	min	max	median	min	max	median	min	max	median	min	max	median	min	max	p
Mo	0.044	0.015	0.504	0.043	<0.006	0.152	0.113	<0.006	0.888	0.643	0.040	2.045	0.032	<0.006	0.392	0.032	0.013	0.046	0.072	0.029	0.561	ns
Nb	<0.0007	<0.0007	0.0035	<0.0007	<0.0007	0.0076	<0.0007	<0.0007	0.0066	<0.0007	<0.0007	0.0085	0.0008	<0.0007	0.0013	0.0009	<0.0007	0.0011	<0.0007	<0.0007	0.0069	nd
Nd	<0.0004	<0.0004	0.0044	<0.0004	<0.0004	0.0016	<0.0004	<0.0004	0.0019	<0.0004	<0.0004	0.0023	<0.0004	<0.0004	0.0064	<0.0004	<0.0004	0.0073	<0.0004	<0.0004	0.0016	nd
Ni	0.13	0.06	0.57	0.19	0.12	1.4	0.065	<0.008	0.14	0.21	0.05	0.58	0.215	0.090	0.500	0.22	0.09	1.57	0.22	0.08	0.41	ns
P	<300	<300	<300	<300	<300	<300	<300	<300	<300	<300	<300	<300	<300	<300	<300	<300	<300	<300	<300	<300	<300	nd
Pb	<0.01	<0.01	0.12	<0.01	<0.01	<0.01	<0.01	<0.01	<0.01	<0.01	<0.01	0.05	0.024	<0.01	0.36	<0.01	<0.01	<0.01	<0.01	<0.01	<0.01	nd
Pr	<0.001	<0.001	<0.001	<0.001	<0.001	<0.001	<0.001	<0.001	<0.001	<0.001	<0.001	<0.001	<0.001	<0.001	<0.001	<0.001	<0.001	<0.001	<0.001	<0.001	<0.001	nd
Rb	<0.02	<0.02	<0.02	<0.02	<0.02	<0.02	<0.02	<0.02	<0.02	<0.02	<0.02	<0.02	<0.02	<0.02	<0.02	<0.02	<0.02	<0.02	<0.02	<0.02	<0.02	nd
Sb	0.005	<0.003	0.199	0.006	<0.003	0.491	<0.003	<0.003	0.069	<0.003	<0.003	0.033	0.011	<0.003	0.049	<0.003	<0.003	0.144	<0.003	<0.003	0.386	ns
Se	<0.1	<0.1	<0.1	<0.1	<0.1	<0.1	<0.1	<0.1	<0.1	<0.1	<0.1	<0.1	<0.1	<0.1	<0.1	<0.1	<0.1	<0.1	<0.1	<0.1	<0.1	nd
Si	<10	<10	34	**26**[a]	<10	50	<10	<10	64	<10	<10	23	**<10**[a]	<10	<10	16	<10	42	<10	<10	46	**<0.05**
Sn	0.00225	<0.0003	0.0182	0.0018	0.0008	0.0047	0.00035	<0.0003	0.0008	0.0031	0.0014	0.0103	0.0053	0.0004	0.0454	0.0037	0.0005	0.0049	0.0020	<0.0003	0.0043	ns
Sr	**1.45**[a]	<0.1	13.9	0.65	<0.1	1.5	**<0.1**[a,b,c]	<0.1	0.30	0.7	<0.1	11.6	0.3	<0.1	3.1	**1.4**[c]	1.1	5.8	**1.4**[b]	0.2	6.6	**a<0.05, b<0.01, c<0.01**
Tb	<0.0001	<0.0001	<0.0001	<0.0001	<0.0001	<0.0001	<0.0001	<0.0001	<0.0001	<0.0001	<0.0001	<0.0001	<0.0001	<0.0001	<0.0001	<0.0001	<0.0001	<0.0001	<0.0001	<0.0001	<0.0001	nd
Te	<0.007	<0.007	<0.007	<0.007	<0.007	<0.007	<0.007	<0.007	<0.007	<0.007	<0.007	<0.007	<0.007	<0.007	<0.007	<0.007	<0.007	<0.007	<0.007	<0.007	<0.007	nd
Ti	<0.04	<0.04	0.12	<0.04	<0.04	2.93	0.23	<0.04	2.59	0.14	<0.04	2.99	<0.04	<0.04	0.90	<0.04	<0.04	0.14	<0.04	<0.04	2.45	ns
Tl	<0.001	<0.001	0.005	<0.001	<0.001	0.014	<0.001	<0.001	0.005	<0.001	<0.001	0.027	0.0065	<0.001	0.058	<0.001	<0.001	0.004	<0.001	<0.001	0.015	nd
U	0.0004	<0.0001	0.0011	0.0012	0.0002	0.0045	<0.0001	<0.0001	0.0069	0.0005	<0.0001	0.008	0.00015	<0.0001	0.0031	0.0010	<0.0001	0.0018	0.0003	<0.0001	0.0013	ns
V	<0.2	<0.2	<0.2	<0.2	<0.2	0.6	<0.2	<0.2	0.8	<0.2	<0.2	0.5	<0.2	<0.2	0.4	<0.2	<0.2	0.3	<0.2	<0.2	0.4	nd
W	0.021	0.006	0.122	0.014	<0.003	0.058	0.011	<0.003	0.066	0.085	<0.003	5.46	0.007	<0.003	1.901	0.019	0.005	0.097	0.015	<0.003	1.15	ns
Zn	<0.3	<0.3	2.30	0.8	<0.3	49	0.55	<0.3	49.1	0.9	<0.3	53.3	1.5	<0.3	12.7	<0.3	<0.3	7.9	<0.3	<0.3	2.0	ns
Zr	0.0175	<0.001	0.279	0.066	0.006	0.686	0.103	<0.001	0.556	0.074	0.005	0.427	**<0.001**[a]	<0.001	0.288	0.034	0.012	0.805	**0.149**[a]	0.002	1.22	**<0.05**

Non-parametric Kruskal–Wallis test for independent samples followed by pairwise comparisons was applied: "-" = not determined; "ns" = not significant at $p > 0.05$; and the data in bold with the same superscript letters within rows were significantly different ($p < 0.05$).

Table S2. Kruskal–Wallis test statistics and Bonferroni-adjusted pairwise comparisons for elements showing significant color-dependent differences in soluble elemental concentrations in commercial tattoo inks.

Element	H[a]	df[b]	Global p-values[c]	Significant post hoc comparisons (adjusted p)[d]
Al	14.152	6	0.028	Black vs Green (0.021)
Si	14.577	6	0.024	Black vs Yellow (0.019)
Cu	39.619	6	0.000	White vs Blue (0.006); White vs Green (0.001); Black vs Blue (0.012); Black vs Green (0.001); Red vs Blue (0.020); Red vs Green (0.001); Orange vs Green (0.010); Yellow vs Green (0.036)
Ga	17.996	6	0.006	White vs Red (0.006)
Sr	23.089	6	<0.001	White vs Red (0.018); White vs Green (0.003); White vs Orange (0.003)
Zr	14.852	6	0.021	Black vs Green (0.029)

[a] H: Kruskal–Wallis test statistic. [b] df: degrees of freedom (number of color groups − 1). [c] Global p-values refer to the overall Kruskal–Wallis test. [d] Pairwise comparisons were performed using Bonferroni correction for multiple testing. Only statistically significant comparisons (adjusted $p < 0.05$) are reported. [e] Adjusted p-values correspond to the "Modified Significance" values generated by SPSS 27.

Table S3. Summary of studies published in the last 10 years on the elemental composition of tattoo inks.

Year	Country	Number of samples tested	Analytical tecnique	Element analyzed	Method	Reference
2018	Denmark, Germany	3	ICP-MS	Cd, Cr, Cu, Fe, Hg, Ni, Pb, Zn	Digestion with HNO_3, and HCl	37
2019	Portugal, Germany	7	AAS, AMA	Cd, Cr, Cu, Hg, Ni, Pb	Total digestion of 0.2 g of ink samples with HNO_3, and H_2O_2	17
2020	Italy	7	ICP-MS (semiquantitative analysis)	Al, As, Ba, Cd, Co, Cr, Cu, Fe, Hg, Mn, Mo, Ni, Pb, Sb, Se, Sn, Ti, Zn	Total digestion with HNO_3, HF, and H_2O_2	18
2021	Sweden, Austria, Canada	73	ICP-MS	Al, As, Ba, Bi, Cd, Co, Cr, Cu, Fe, Hg, Mn, Mo, Ni, Pb, Pd, Sb, Sn, Sr, W, Zn	Total (0.1 g of ink samples by microwave assisted digestion with HNO3) and water-soluble (0.5 g of ink samples extracted in 10 mL 0.9% NaCl for 12 h) elements	25
2023	Turkey	9	ICP-MS	Al, Ni, Cu, Co, Fe, Zn	Total digestion of 0.3 g of ink sample with 6 mL of HNO_3, 3 mL of HCl, and 0.8 mL of HF	19
2023	Italy	16	ICP-OES, ICP-MS	As, Ba, Cd, Co, Cr, Cu, Fe, Hg, Mn, Mo, Ni, Pb, Sb, Se, Sn, Sr, V, Zn	Total digestion of 0.25 g of ink sample with 1 mL of HCl and 1 mL of H_2O_2. The residue was then treated with 1 mL of HF and 4 mL of HNO_3 and heated on a hot plate until the sample was completely mineralized.	38
2024	Turkey	9	ICP-MS	Cd, Cr, Hg, Pb	Total digestion with HNO_3, HCl, and HF in the ratio 6:3:0.8	39
2026	Australia	15	ICP-MS	Ag, Al, As, B, Ba, Be, Bi, Ca,	Samples were first diluted 1:1000 in	40

				Cd, Ce, Co, Cr, Cs, Cu, Dy, Er, Eu, Fe, Ga, Gd, Ge, Hf, Hg, Ho, In, K, La, Li, Lu, Mg, Mn, Mo, Na, Nb, Nd, Ni, P, Pb, Pr, Rb, Re, Sb, Sc, Se, Si, Sm, Sn, Sr, Ta, Tb, Te, Ti, Tl, Tm, U, V, W, Y, Yb, Zn, Zr.	ultrapure water and mixed thoroughly, then further diluted 1:5 with 2.5 % HNO_3 to yield a final concentration of 2 % HNO_3.	
This work	Italy		ICP-MS	Al, As, B, Ba, Be, Bi, Ca, Cd, Ce, Co, Cr, Cs, Cu, Dy, Fe, Ga, Gd, K, La, Li, Mg, Mn, Mo, Nb, Nd, Ni, P, Pb, Pr, Rb, Sb, Se, Si, Sn, Sr, Tb, Te, Ti, Tl, U, V, W, Zn, Zr	250 mg of ink samples were extracted with 5 mL of artificial sweat and incubated at 37 °C for 1 h in a thermostated water bath	-

Abbreviations: ICP, inductively coupled plasma; MS, mass spectrometry; OES, optical emission spectroscopy; AAS, atomic absorption spectrometry; AMA, automatic Hg analyzer.
